# Proposed New AV-Type Test-Bed for Accurate and Reliable Fish-Eye Lens Camera Self-Calibration

**DOI:** 10.3390/s21082776

**Published:** 2021-04-14

**Authors:** Kang Hyeok Choi, Changjae Kim

**Affiliations:** 1 Korea Institute of Civil Engineering and Building Technology, 283 Goyangdae-Ro, Ilsanseo-Gu, Goyang-Si 10223, Gyeonggi-Do, Korea; cwsurgy@kict.re.kr; 2 Department of Civil and Environmental Engineering, Myongji University, 116 Myongji-Ro, Cheoin-Gu, Yongin 17058, Gyeonggi-Do, Korea

**Keywords:** fish-eye lens camera, self-calibration, interior orientation parameters, calibration test-bed

## Abstract

The fish-eye lens camera has a wide field of view that makes it effective for various applications and sensor systems. However, it incurs strong geometric distortion in the image due to compressive recording of the outer part of the image. Such distortion must be interpreted accurately through a self-calibration procedure. This paper proposes a new type of test-bed (the AV-type test-bed) that can effect a balanced distribution of image points and a low level of correlation between orientation parameters. The effectiveness of the proposed test-bed in the process of camera self-calibration was verified through the analysis of experimental results from both a simulation and real datasets. In the simulation experiments, the self-calibration procedures were performed using the proposed test-bed, four different projection models, and five different datasets. For all of the cases, the Root Mean Square residuals (RMS-residuals) of the experiments were lower than one-half pixel. The real experiments, meanwhile, were carried out using two different cameras and five different datasets. These results showed high levels of calibration accuracy (i.e., lower than the minimum value of RMS-residuals: 0.39 pixels). Based on the above analyses, we were able to verify the effectiveness of the proposed AV-type test-bed in the process of camera self-calibration.

## 1. Introduction

In recent years, fish-eye lens cameras have been used in various fields including indoor and outdoor 3D modeling, autonomous driving, augmented and virtual reality, Simultaneous Localization And Mapping (SLAM), people- and motion-detection, and so on. This camera has a wider Field Of View (FOV) relative to a conventional optical camera; hence, it can record an image over a much wider area. Campos et al. [1] used a backpack-mounted fish-eye lens camera for forest mapping, and Schöps et al. [2] utilized fish-eye lens cameras in the production of a three-dimensional model of a large area. Ronchetti et al. [3] employed a drone-mounted fish-eye lens camera to produce a very-high-resolution Digital Terrain Model (DTM) for precision agriculture. Zia et al. [4] proposed a UAV sensor system to produce a high-quality 360-degree panorama, and used a fish-eye lens camera for securing sufficient overlap between adjacent camera views. Kim et al. [5] incorporated two fish-eye cameras into the design of an obstacle detection system without blind spot at the rear of the car. Perez-Yus et al. [6] overcame the narrow FOV of an RGB-depth system by coupling a fish-eye camera to it, and Sreedhar et al. [7] proposed a system for Virtual Reality (VR) applications using multiple fish-eye lenses mounted on a camera system covering the entire 360-degree FOV. Sánchez et al. [8] improved the accuracy of urban navigation using a fish-eye lens camera. Xu et al. [9] used a fish-eye lens camera for production of a 3D motion capture system, and Krams et al. [10] used a fish-eye lens camera for people-detection purposes.

The fish-eye lens camera has an obvious advantage relative to the conventional perspective projection camera; however, it also has a disadvantage that should be recognized. The fish-eye lens camera shows strong geometric distortion in the image, due to compressively recording the outer part of the image relative to the center part. Hence, such distortion must be corrected through the self-calibration procedure [11,12], particularly when the camera is used in fields where image geometry is important. Moreau et al. [13] used a stereoscope composed of fish-eye lens cameras in order to increase GNSS localization accuracy for vehicles. For this, they performed the self-calibration procedure. Marković et al. [14] and Caruso et al. [15] used pre-calibrated fish-eye lens cameras for SLAM. Auysakul et al. [16] proposed the Around View Monitor (AVM) system for the Advanced Driving Assistance System (ADAS) using fish-eye lens cameras, all of which were rectified through self-calibration. Gao et al. [17] and Yang et al. [18] derived a driving assistance system consisting of pre-calibrated fish-eye lens cameras. Nakazawa et al. [19] proposed an indoor positioning method based on Visible Light Communication (VLC) that utilized a fish-eye lens camera to detect additional LED lights and, therefore, improve the positioning accuracy. Gabor et al. [20] proposed a sensor system based on pre-calibrated multiple fish-eye lens cameras to accurately estimate the 3D positions and motions of clouds. Berveglieri et al. [21] used a fish-eye lens camera for automated mapping and measurement of tree stems, preparatorily having performed self-calibration of the camera. It should be emphasized that all of the studies mentioned above employed camera self-calibration procedures or used pre-calibrated fish-eye lens cameras for their applications in order to achieve high positioning accuracies.

### 1.1. Previous Studies of Fish-Eye Lens Camera Self-Calibration

Self-calibration is a process of determining Interior Orientation Parameters (IOPs) that are used to interpret the geometry of sensory data recording. The process estimates the internal characteristics (i.e., principal point coordinates, focal length, and lens distortion parameters) of a camera considered. The mathematical model of imaging geometry of an optical camera includes such characteristics [22]. Hence, precision of camera self-calibration can significantly affect the quality of the mathematical interpretation of imaging geometry. In other words, accurately estimated IOPs can correct the geometric distortions of the images recorded.

The mathematical model of imaging geometry of an optical camera is represented by two components, which are lens distortion and the projection models. Lens distortion model has been verified through various previous studies of conventional [23,24,25,26,27] and macro [28] lenses while considering configurations of test-beds, specifications of cameras (e.g., FOV, focal length, etc.), and correlations between parameters. Lens distortion is a phenomenon shown in all optical lenses, and a lens distortion model can be used for not only the conventional and macro lens but also the fish-eye lens [29]. On the other hand, the fish-eye lens camera has different projection models compared with conventional and macro cameras. The equidistant, equisolid-angle, orthogonal, and stereographic projection models have been considered as the typical basic projection models of the fish-eye lens camera [30,31,32,33]. Previous studies demonstrated that these basic models can adequately explain the geometric distortion of a fish-eye lens through the self-calibration procedure. Hughes et al. [34,35] proposed a self-calibration method using the extracted vanishing points from test images, and performed self-calibration for an equidistant projection fish-eye lens camera. Sahin [36] suggested a self-calibration process for a fish-eye lens camera and verified the process by applying the equidistant projection model. Bakstein et al. [37] performed self-calibration based on a projection model that combines equisolid-angle with stereographic projection. Schneider et al. [38] performed self-calibration of a fish-eye lens camera by applying the typical basic fish-eye lens projection models: equidistant, equisolid-angle, orthogonal, and stereographic. The authors demonstrated that the distortion of a fish-eye lens camera could be accounted for and corrected using a combination of the typical projection and lens distortion models, regardless of which specific projection model is used.

Moreover, basic fish-eye projection models have been adopted and verified through various application-based experiments, researchers having developed their own sensor systems with fish-eye lens cameras and performed camera self-calibration procedures. Yang et al. [18] performed self-calibration based on an equidistant projection model for a driving assistance system. Nakazawa et al. [19] and Berveglieri et al. [21] calibrated an equidistant projection camera using stripe patterns and coded targets, respectively. Li et al. [39] performed self-calibration based on the equidistant projection model in order to develop a spherical image sensor using fish-eye lens cameras. Chunyan et al. [40] calibrated fish-eye lens cameras used as hazard cameras for a lunar rover by applying the equidistant projection model. Schneider et al. [41] produced a multi-sensor system using lidar and a fish-eye lens camera, calibrating the fish-eye lens camera by means of equisolid-angle projection. Perfetti et al. [42] conducted 3D modeling using equisolid-angle and stereographic projection lens cameras for narrow and hypogea environments, and calibrated both fish-eye lens cameras.

Other models not belonging to the basic projection model family have also been proposed based on several studies. Basu and Licardie [43] proposed and verified Fish-Eye Transform (FET) and Polynomial Fish-Eye Transform (PFET). Devernay et al. [44] and Fitzgibbon [45] proposed Field Of View (FOV) and division models, respectively. Hughes et al. [29] proposed a self-calibration method and compared its accuracy using equidistant, equisolid-angle, orthogonal, stereographic, FET, PFET, and FOV models.

The previous studies relevant to fish-eye lens self-calibration mostly have either verified the fitness of the projection models to the imaging geometry or compared to the performances of models with each other. Moreover, they generally performed self-calibration without analyzing the effects of the test-bed on the calibration quality. Most of them, in fact, simply used the same test-bed as used for perspective projection camera calibration. More specifically, they used either (i) test-beds using a single plane [6,11,16,17,18,34,35,42,46,47,48] or (ii) test-beds using two or three planes [12,21,30,33,39,44]. Test-bed shape affects the correlation between the orientation parameters; consequently, the level of correlation significantly affects the levels of accuracy and reliability of the estimated parameters [26,49,50]. In short, many studies have underestimated the effect of the test-bed on the quality of fish-eye lens camera calibration.

In a few studies [36,38,41,51], several test-beds were proposed for accurate self-calibration; however, they were mostly for the balanced distribution of image points, not for the reduction of parameter-correlations. More specifically, Sahin [36] used satellite antennas of 1.5-m diameter to establish a test-bed. By using the test-bed, the author balanced the distribution of the image points and also established different depths. Schneider et al. [38,41] and Schwalbe [51] established a calibration room in which ground control points were established in one half of the room in a way that all the points could be well-distributed.

On the other hand, Choi et al. [50] found that according to test-bed shapes, the accuracies of fish-eye lens camera self-calibration showed different levels. They demonstrated the advantages and disadvantages of four different test-beds, and proposed a self-calibration method for accurate estimation of IOPs. However, this study has the following limitations. Firstly, the analysis was performed based on simulation experiments only, and the findings were not re-verified using real data. Secondly, the V-type test-bed, which significantly reduces parameter-correlations, is not easily established and kept semi-permanently in an indoor environment. Such limitations need to be overcome in order to make accurate and reliable calibration solutions possible.

### 1.2. Purpose of Study

The objectives of this study were (1) to propose a new type of test-bed that is suitable for self-calibration of a fish-eye lens camera in the indoor environment, and (2) to verify the effectiveness of the proposed test-bed through both simulation and real experiments.

The test-bed herein proposed is based on the findings of Choi et al. [50]. According to their study, the A-type test-bed has an advantage in terms of the explanation of the lens distortion phenomenon, since it has a high coverage ratio for image points. Also, A-type objects (e.g., concave corners) can be easily found in the indoor environment. However, such a test-bed does not provide stable calibration solutions due to the high correlation between some of the orientation parameters. On the other hand, the V-type test-bed works well in resolving the correlation issue; however, it has a weak point in terms of the coverage ratio of image points. Also, it is hard to find V-type objects (e.g., convex corners) in the indoor environment.

In the present study, a new type of test-bed was designed to reconcile and compensate for the pros and cons of the A- and V-type test-beds. In other words, the test-bed was designed to effect (i) a balanced distribution of image points and (ii) a low level of correlation between orientation parameters. The effectiveness of the proposed test-bed in the process of camera self-calibration was verified through analysis of the experimental results from simulation and real datasets. The experimental results were evaluated based on the accuracy of principal point coordinates and focal length as well as how well the lens distortion parameters interpreted the distortion caused by a fish-eye lens. The simulation-based camera self-calibration process was carried out before conducting the process using real data. The simulation experiments were performed for the following advantages. With simulations, perfect control of all involved parameters is convenient, and the accuracy of results can be clearly confirmed. In other words, direct comparison between the estimated parameters values and true ones is possible in the simulated environment. In the present study, following the simulation experiments, a new type of test-bed was set up in an indoor environment, and self-calibration was carried out. The real experiments were necessary in order to fully prove the validity of the fish-eye lens self-calibration approach using a new type of test-bed suggested in this research.

This paper describes the present research contents in the following order. Section 2 describes the mathematical model of the fish-eye lens camera. Section 3 introduces the proposed test-bed and explains the design of the simulation and real experiments. Section 4 analyzes the experimental results as derived from the simulation and real datasets. Section 5 provides a discussion of the proposed method in terms of contributions, while comparing it with previous studies. Finally, Section 6 draws conclusions.

## 2. Mathematical Model of Fish-Eye Lens Camera

This section, first, introduces the projection models of the fish-eye lens camera. The representative four different projection models (i.e., equidistant, equisolid-angle, orthogonal, and stereographic projection) of the fish-eye lens camera are explained. Afterward, the well-known lens distortion model, which applies for the four projection models, is introduced.

### 2.1. Projection Model of Fish-Eye Lens Camera

**Figure 1:**
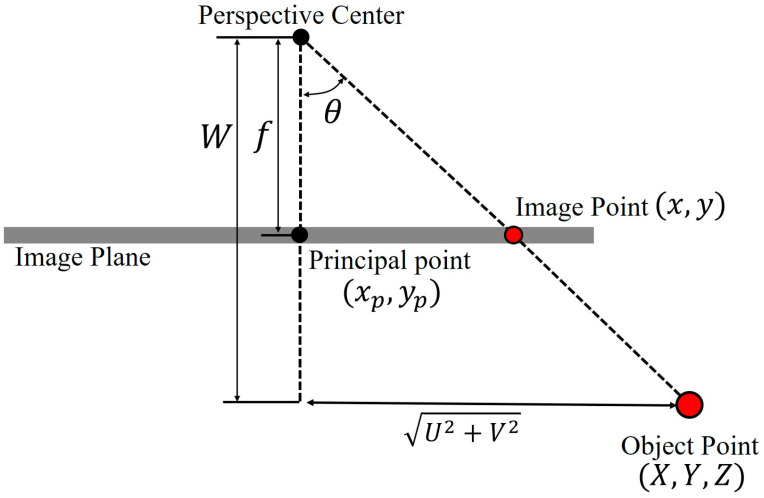
Involved parameters in imaging geometry.

The projection model of the fish-eye lens camera is represented by IOPs, Exterior Orientation Parameters (EOPs), coordinates of image points, and coordinates of object points. The model is given by Equations (1)–(9) and Figure 1, where (*x, y*) and ($x_{p}$, $y_{p}$) are the coordinates of the image and principal point respectively; r is the distance between the principal and the image point; (*X, Y, Z*) are the coordinates of the object point; (*X*0, *Y*0, *Z*0, *ω, φ, κ*) are EOPs; *M(ω, φ, κ)* is a three-dimensional rotation matrix; f is the focal length, and *θ* is the incident angle of the object point:

(1) $$x=x_{p}-\frac{r}{\sqrt{U^{2}+V^{2}}}U$$

(2) $$y=y_{p}-\frac{r}{\sqrt{U^{2}+V^{2}}}V$$

(3) $$\left[\begin{array}{c} U\\ V\\ W \end{array}\right]=M\left(\omega ,\varphi ,\kappa \right)\left[\begin{array}{c} X-X0\\ Y-Y0\\ Z-Z0 \end{array}\right]$$

(4) If Perspective Projection (Normal lens)  r=ftanθ

(5) Else if Equidistant Projection (Fish−eye lens)  r=fθ

(6) $$\mathrm{Else}\;\mathrm{if}\;\mathrm{Equisolid}-\mathrm{angle}\;\mathrm{Projection}\;\left(\mathrm{Fish}-\mathrm{eye}\;\mathrm{lens}\right)\;\;r=f2\sin\frac{\theta }{2}$$

(7) Else if Orthogonal Projection (Fish−eye lens)  r=fsinθ

(8) $$\mathrm{Else}\;\mathrm{if}\;\mathrm{Stereographic}\;\mathrm{Projection}\;\left(\mathrm{Fish}-\mathrm{eye}\;\mathrm{lens}\right)\;\;r=f2\tan\frac{\theta }{2}$$

(9) $$\theta =arctan\;\left(\frac{\sqrt{U^{2}+V^{2}}}{W}\right)$$

### 2.2. Lens Distortion Model

The lens distortion model is represented by Equations (10)–(13). Where Δ*x* and Δ*y* are distortions of the image coordinates *x* and *y*, respectively; (*K*_1_, *K*_2_, *K*_3_) are the radial lens distortion parameters; (*P*_1_, *P*_2_) are the decentering distortion parameters; and (*A*_1_, *A*_2_) are the in-plane distortion parameters. Recall that r is the distance between the principal and the image point.

Equations (14) and (15) are the final mathematical models of the fish-eye lens camera, and are expressed using the projection and lens distortion models. The mathematical model includes projection model Equations (1) and (2) along with distortion model Equations (10) and (11).

(10) $$\Delta x=\bar{x}\left(K_{1}r^{2}+K_{2}r^{4}+K_{3}r^{6}\right)+P_{1}\left(r^{2}+2{\bar{x}}^{2}\right)+2P_{2}\bar{x}\bar{y}+A_{1}\bar{x}+A_{2}\bar{y}$$

(11) $$\Delta y=\bar{y}\left(K_{1}r^{2}+K_{2}r^{4}+K_{3}r^{6}\right)+2P_{1}\bar{x}\bar{y}+P_{2}\left(r^{2}+2{\bar{y}}^{2}\right)$$

(12) $$\bar{x}=x-x_{p}$$

(13) $$\bar{y}=y-y_{p}$$

(14) $$x=x_{p}-\frac{r}{\sqrt{U^{2}+V^{2}}}U+\Delta x$$

(15) $$y=y_{p}-\frac{r}{\sqrt{U^{2}+V^{2}}}V+\Delta y$$

## 3. Camera Calibration Design: Simulation and Real Experiments

This study undertook to derive a new type of test-bed for efficient and accurate fish-eye lens self-calibration in the indoor environment. The test-bed (called the AV-type test-bed in this research) was designed by combining the A-and V-type components. Usually, the A- (e.g., concave corner) and V-type (e.g., convex corner) components are seen inside and outside of buildings, respectively. The AV-type test-bed was proposed to adopt the advantages of both the A- and V-type test-beds, as mentioned in Section 1.2. In other words, the authors planned to use the test-bed so as to be able to explain the lens distortion phenomenon and effectively reduce the level of correlation between the orientation parameters at the same time.

Calibration experiments were designed in two parts: simulations and real experiments. The simulation experiments proceeded according to the following steps: (i) AV-type test-bed shape design, (ii) camera specification setting and projection model selection, (iii) simulation images generation, and (iv) camera self-calibration and result analysis. Four different projection models were utilized for the data generation and camera self-calibration procedures. As for the real experiments, they proceeded according to the following steps: (i) real AV-type test-bed installation, (ii) image acquisition using real fish-eye cameras, and (iii) camera self-calibration and result analysis. In both the simulation and real experiments, the various analyses were carried out while checking the stability of self-calibration and the accuracy of the IOPs.

### 3.1. Design of Simulation Experiments

**Figure 2:**
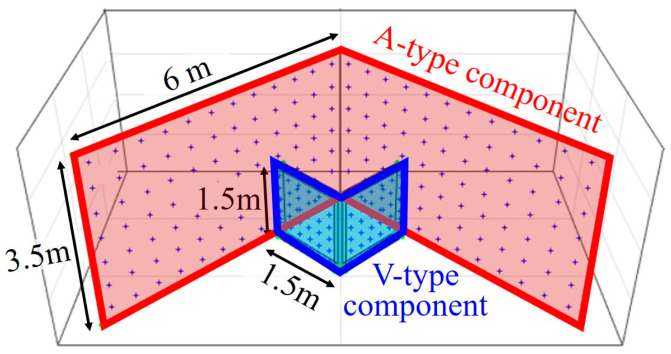
Designed AV-type test-bed for simulation experiments.

The simulation experiments were carried out to confirm that the proposed AV-type test-bed is appropriate for explaining lens distortions and reducing the correlation between orientation parameters in consideration of the four different projection models. The design of test-bed applied in the simulation experiments is shown in Figure 2. The A-type component was designed with two planes (each plane 3.5 m in height and 6 m in width). The V-type component was designed with two smaller planes (each plane 1.5 m in height and 1.5 m in width), and was positioned in the middle of the A-type component.

**Figure 3:**
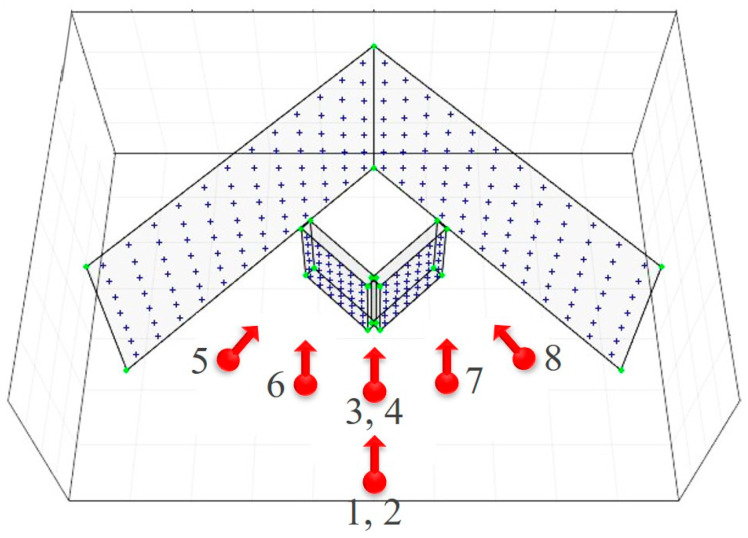
Configuration of image acquisition for simulations.

The specifications of the fish-eye lens camera sets for the simulations are provided in Table 1. These values were predetermined by similarly following a Sunex DSL31 fish-eye lens (Sunex, Carlsbad, CA, USA) and a Chameleon3 USB3 5.0 MP camera body (FLIR, Wilsonville, OR, USA). Figure 3 shows the configuration of the image acquisition for the simulations. Using the AV-type test-bed, eight images were produced in different shooting positions and viewing angles. The simulation image data included both landscape (*k* = 0°) and portrait (*k* = 90°) images for de-correlation between the principal point coordinates and EOPs. Location numbers 1 and 2 had the same shooting position but different viewing angles (i.e., *k* angles). The same was true for location numbers 3 and 4. More specifically, location numbers 2 and 4 were set to take portrait images, and location numbers 1, 3, and 5 to 8 were for landscape images. Then, the simulated image datasets were prepared by using the AV-type test-bed, camera specification, image acquisition configuration, and projection model selected.

Self-calibrations were performed using five different simulation datasets for each projection model. Table 2 shows the different cases of simulation datasets (dataset a-e) utilized for the self-calibrations. The datasets were made of two, four, or eight images including at least one portrait image to resolve the correlation between the orientation parameters (especially, $x_{p}-X0$, $y_{p}-Y0$). Datasets a, b, c, d, and e were used for the evaluation of the AV-type test-bed.

### 3.2. Design of Real Experiments

**Figure 4:**
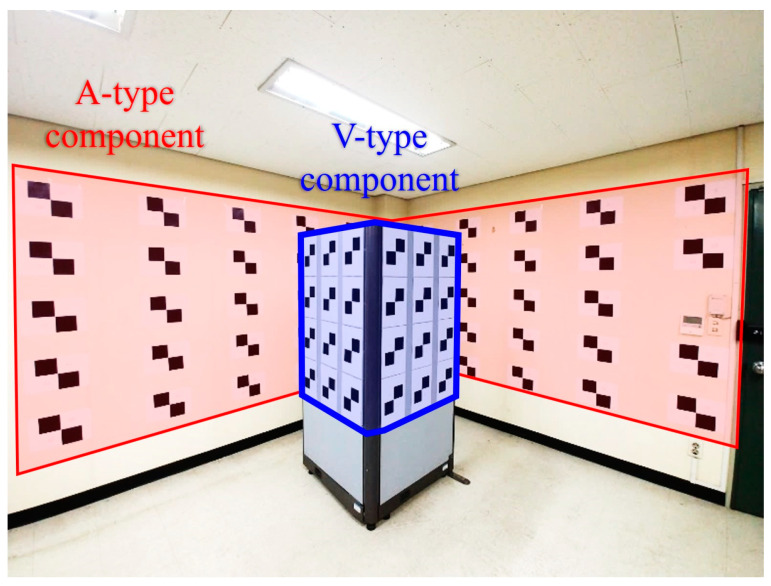
Established test-bed for real experiments.

In the real experiments, the proposed AV-type test-bed was produced as shown in Figure 4. The corner of the room was used as the A-type component consisting of two walls sized about 2 m in height by 3 m in width. The additional planes (actually, office partitions) were used to build the V-type component. One partition plane’s size was about 1.2 m in height by 0.9 m in width.

Two fish-eye lens cameras of different projection models were used in the real experiments. Table 3 shows the employed fish-eye lens cameras and their specifications. Fish-eye lens cameras 1 and 2 were equidistant and equisolid-angle projection cameras, respectively.

**Figure 5:**
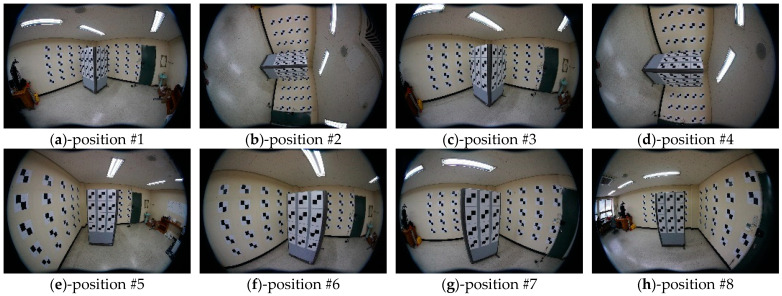
Images of camera 1 (equidistant).

**Figure 6:**
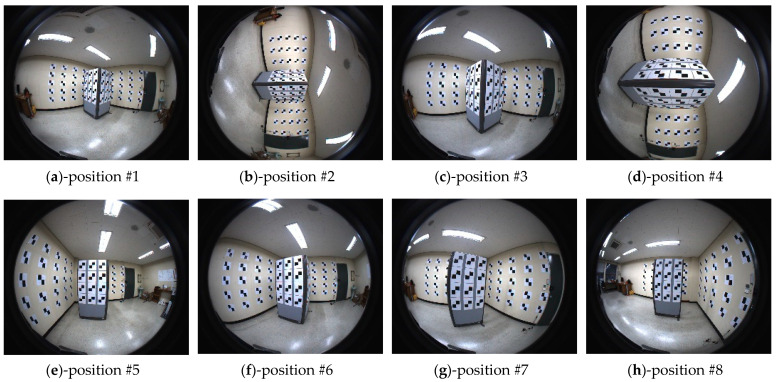
Images of camera 2 (equisolid angle).

Table 4 shows the different cases of real datasets utilized for the self-calibrations. The configuration of the image acquisition and the dataset for the real experiments were similar to the simulation cases (in Table 2). Datasets A, B, C, D, and E were acquired using the AV-type test-bed and applied to fish-eye lens camera self-calibration. Figure 5 and Figure 6 show the images taken by cameras 1 and 2, respectively.

## 4. Results of Self-Calibration

The analysis of the experimental results proceeded in the same sequence as for the simulation and real experiments. Each experiment was analyzed in terms of three aspects. Firstly, it was determined whether the proposed test-bed contributed to de-correlation between orientation parameters. If the correlation issue is resolved, self-calibration performs in stable states without divergence or a local minimum problem. Secondly, it was confirmed whether the estimated IOPs were accurate. In the cases of the simulation experiments, the estimated IOPs were directly compared with true IOPs that had been pre-set. In the cases of the real experiments, the accuracy of the estimated IOPs was evaluated indirectly by checking the residuals of the image points and the standard deviation of the orientation parameters.

### 4.1. Experimental Results Using Simulation Datasets

Camera self-calibration was carried out to confirm the effectiveness of the proposed AV-type test-bed regardless of projection models. The simulation-based experiments were analyzed in two steps: the first step evaluated the stability and correlation of the solution; the second step analyzed the accuracies of the estimated IOPs.

#### 4.1.1. Stability and Correlation Analysis in Simulation Experiments

The stability and correlation analysis were carried out based on the results of the simulated experiments. Stability was evaluated in two statuses, which were ‘stable’ and ‘unstable’. When most of the orientation parameters diverged, or when the solution did not reach the global minimum, stability was evaluated as “unstable”. On the other hand, stability was evaluated as “stable” when the parameters converged while reaching the global minimum. Table 5 shows the results of the stability and correlation analyses.

The correlation values from the five different datasets and their mean values are shown. All of the mean values of the projection models were lower than 0.72. Medium or somewhat high correlation values were shown in case of f−Z0. The highest correlation values in the cases of f−Z0 for all projection models occurred when the dataset a, which was composed of just two images, was used. It should be noted that all of the self-calibration solutions stably converged even though some cases (i.e., dataset a) had somewhat (not significantly) high correlations. The stabilities and accuracies of the self-calibration solutions are proven also in the following Section 4.1.2.

#### 4.1.2. Accuracy of IOPs in Simulation Experiments

The absolute errors of the principal point coordinates and focal length were calculated by comparing the estimated values with the pre-set ones (shown in Table 1 for the simulation). Table 6 and Table 7 show the absolute errors of the principal point coordinates and focal length, respectively. The largest errors of the principal point coordinates and focal length were 0.34 and 0.56 pixels, respectively. These indicate that both the principal point coordinates and focal length were estimated to high accuracies.

The accuracy of the lens distortion parameters was evaluated by comparing the true and the estimated distortions. The distortion was calculated at all pixels either using the pre-set or the estimated parameters. Table 8 shows the Root Mean Square Error (RMSE) values of lens distortion. The largest RMSE value was 0.68 pixels, which indicates that the estimated distortion parameters had high accuracies regardless of the dataset utilized.

Table 9 shows the RMS-residuals calculated using all of the estimated IOPs (i.e., the principal point coordinates, focal length, and lens distortion parameters). The largest RMS-residual was 0.46 pixels, which also indicates that the estimated IOPs had high accuracies.

Based on the accuracy analysis of IOPs using the simulated datasets, it was verified that the proposed AV-type test-bed is appropriate to use for self-calibration of a fish-eye lens camera. Principal point coordinates, focal length, and distortion parameters were estimated accurately by using the proposed test-bed. The accuracy of the IOPs was high, even for the case of ‘Dataset a’ composed of just two images. In other words, by using the AV-type test-bed, self-calibration for the fish-eye lens cameras could be performed accurately and efficiently.

### 4.2. Experimental Results Using Real Datasets

The effectiveness of the proposed AV-type test-bed in the process of camera self-calibration was re-verified by analyzing the results of the real experiments. Similarly to the simulation case, the real experiments were analyzed in two steps. In the first step, the correlations between orientation parameters were evaluated. In the second step, the accuracy of the estimated IOPs was evaluated using the standard deviations and RMS-residuals of image points.

#### 4.2.1. Stability and Correlation Analysis in Real Experiments

All of the self-calibrations using the two fish-eye lens cameras, five datasets and proposed AV-type test-bed were performed without divergence or local minimum problem. This means that the implemented self-calibrations were all stably converged. More specifically, Table 10 shows the correlation values between the orientation parameters derived from the self-calibration results. As seen in the table, most cases (i.e., $x_{p}-X0$, $y_{p}-Y0$, X0−φ, Y0−ω) showed low correlations. On the other hand, relatively medium or high correlation values were shown in the case of f−Z0. The highest correlation values in the cases of f−Z0 for cameras 1 and 2 were 0.95 and 0.93, respectively. These two highest correlations occurred when Dataset A, composed of just two images, was used. It should be noted that all of the self-calibration solutions stably converged even though some cases had somewhat (not significantly) high correlations. The stabilities and accuracies of the self-calibration solutions are proven also in the following Section 4.2.2.

#### 4.2.2. Accuracy of IOPs in Real Experiments

The accuracy of the IOPs was evaluated by checking (i) the estimated IOPs and their standard deviations, and (ii) their RMS-residuals. Firstly, the different IOPs from the five datasets were compared with each other; and the precisions of the parameters were analyzed by checking the standard deviations themselves and by comparing them with the parameter values. Secondly, the RMS-residuals of IOPs showed differences between the measured and estimated image point coordinates. Hence, the accuracy of the estimated IOPs were analyzed via the RMS-residuals.

Table 11 shows the estimated values and standard deviations of the principal point coordinates and focal lengths. For each camera, the principal point coordinates and focal length were estimated as similar values regardless of the dataset used. In the case of camera 1, the maximum absolute differences of $x_{p}$, $y_{p}$, and f among the different datasets were 0.91, 0.74, and 0.37 pixels, respectively. In this case, all of the differences were less than one pixel. In the case of camera 2, the maximum absolute differences of $x_{p}$, $y_{p}$, and f among the different datasets were 0.13, 0.28, and 0.39 pixels, respectively. In this case, all of the differences were less than one-half pixel. In terms of standard deviations, the values themselves were all lower than one-half pixel. Also, all of the standard deviations were much lower than the estimated parameter values.

Table 12 and Table 13 show the estimated distortion parameters and their standard deviations for cameras 1 and 2, respectively. As can be seen, the maximum absolute differences of the lens distortion parameters among the different datasets were almost zero. Also, all of the standard deviations were much lower than the corresponding distortion parameter values.

Table 14 shows the RMS-residuals of the IOPs for each dataset and camera. The maximum value of RMS-residuals was 0.39 pixels (lower than one-half pixel). The mean values for cameras 1 and 2 were 0.27, and 0.35 pixels, respectively. Based on the analysis results as tabulated by Table 11, Table 12, Table 13 and Table 14, it was confirmed that the principal point coordinates, focal length, and lens distortion parameters for the two different fish-eye lens cameras had been estimated reliably and accurately.

In this study, based on simulations and real experiments, the effectiveness of the proposed AV-type test-bed was evaluated using different projection models and datasets. The results can be summarized as follows:

- The proposed AV-type test-bed was effective in resolving the correlation between the orientation parameters, and self-calibration was performed stably.
- At the same time, lens distortion was interpreted accurately due to the proposed test-bed having contributed to the balanced distribution of image points.
- The estimated IOPs using the AV-type test-bed showed high accuracy and precision. Even though self-calibration was performed using a dataset composed of just two images, the IOPs were estimated reliably and accurately.

## 5. Discussion

In this section, the effectiveness of the proposed approach is deeply discussed in terms of distribution of image points, and accuracy by comparing it with previous studies. First, a comparative analysis between Choi et al. [50]’s study and the proposed one is carried out based on the experimental results using simulation datasets.

**Figure 7:**
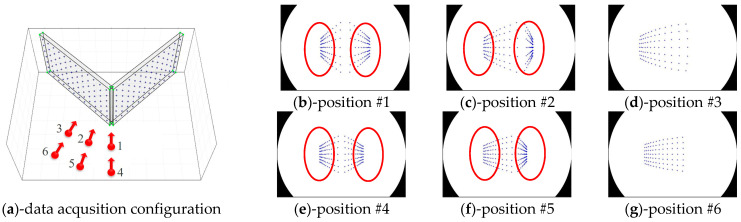
Distributions of image points in case of V-type test-bed (equidistant).

**Figure 8:**
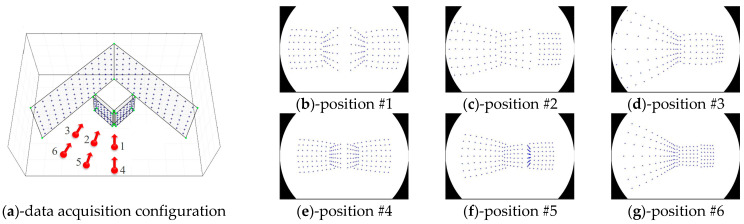
Distributions of image points in case of AV-type test-bed (equidistant).

To compare the distributions of image points according to the utilized types of test-beds, we produced simulation images while changing the data acquisition configuration. Figure 7 shows the simulation images produced by utilizing V-type test-bed, which was selected for self-calibration by Choi et al. [50]. Also, Figure 8 shows the simulation images produced by utilizing AV-type test-bed, which was proposed in this study. One should note that the data acquisition configurations for Figure 7 and Figure 8 are the same.

In terms of the distribution of image points, AV-type test-bed produced relatively well-distributed image points compared to V-type test-bed, as seen in Figure 7 and Figure 8. Especially, the portions enclosed by red lines in Figure 7 show a high density of image points. Such distribution makes the image point measurement difficult in reality and might cause inaccurate lens distortion parameters. To avoid this issue when the V-type test-bed is used for self-calibration, data acquisition configuration should be determined very carefully. On the other hand, we do not see such a high density of image points when the AV-type test-bed is used as seen in Figure 8. Moreover, AV-type test-bed can be easily set up in an indoor environment (such as a corner of a room) and used semi-permanently.

In terms of accuracy, the absolute error of principal point coordinates, absolute error of focal length, RMSE value of lens distortion, and RMS-residuals of IOPs, which are derived from both of the proposed approach and Choi et al. [50]’s study, are compared in Table 15, Table 16, Table 17 and Table 18. In these tables, the number of images utilized for the self-calibration is shown in the parenthesis. When comparing the values for the same projection model, most of the values from the proposed approach were lower than the ones from Choi et al. [50]’s study in all the tables. The mean values calculated for each projection model were, also, compared to each other. The values from the AV-type test-bed were all lower than the ones from the V-type test-bed. This means that the self-calibration performances using AV-type test-bed (even though a smaller number of images are utilized) were superior to the ones using V-type test-bed.

Secondly, additional comparative analysis between the previous studies and the proposed one is carried out based on the experimental results using real datasets. At this stage, one should note that a direct comparison of the performance among different approaches is almost impossible since their type of cameras, projection models, calibration methods, datasets, and environments are all different. Nevertheless, the comparison in Table 19 shows the overall performances of the approaches. In the table, the values of standard deviations, and RMS-residuals of IOPs derived from different approaches are compared to each other.

As seen in the table, it is not easy to directly compare Marcato et al. [12]’s study with the proposed one since they are dealing with different projection models. However, we can, overall, see similar performances in terms of standard deviations. In the case of RMS-residuals of IOPs, the proposed one showed better performance. Marcato et al. [12], however, used many images (i.e., 43 images) for the calibration process compared to the proposed one (i.e., 2–8 images). Sahin [36]’s study showed worse results for both standard deviations and RMS-residuals of IOPs compared to the proposed one. In the case of Schneider et al. [38], it showed worse results for standard deviations; however, a similar result for RMS-residuals of IOPs compared to the proposed approach. The overall comparison through Table 19 proved that even though the proposed approach used a smaller number of images (2 to 8 images), it provided similar or better performances compared to other approaches (which used 9 to 43 images) for the calibration procedure.

## 6. Conclusions

This paper proposed a new type of test-bed (i.e., the AV-type) for stable and accurate self-calibration of a fish-eye lens camera in an indoor environment. The effectiveness of the proposed test-bed was verified through simulation and real experiments. This study was conducted in two steps: (i) camera calibration design, and (ii) validation through simulation and real experiments.

The proposed AV-type test-bed was designed to contribute a balanced distribution of image points (the advantage of the A-type test-bed) along with de-correlation between orientation parameters (the advantage of the V-type test-bed) simultaneously. In addition, the proposed test-bed could be installed conveniently in the indoor space and used semi-permanently.

In the simulation experiments, the self-calibration procedures were performed using the proposed test-bed, four different fish-eye lens projection models, and five different datasets. For all of the cases, the self-calibration proceeded stably, and eventually, provided accurately estimated IOPs. The RMS-residuals of the estimated IOPs were lower than the random noise level (i.e., pre-set to 0.5 pixels).

The real experiments were carried out to re-verify the effectiveness of the proposed AV-type test-bed in the process of camera self-calibration using two different fish-eye lens cameras and five different datasets. All of the real experimental cases showed high levels of calibration accuracy (i.e., lower than the minimum value of RMS-residuals: 0.39 pixels) and precise standard deviations. Through analysis of the data obtained from the simulation and real experiments, we came to the conclusion that the proposed AV-type test-bed was appropriate for self-calibration of the fish-eye lens camera and accurate IOP estimation.

This study will contribute to fish-eye lens camera self-calibration in the following ways. By using the proposed test-bed, it is ensured that the self-calibration of a fish-eye lens camera will be performed in a stable state and that the IOPs will be derived accurately. Also, the proposed test-bed will provide for efficiency of self-calibration in terms of both test-bed installation and operation.

## Figures and Tables

**Table 1:** Specification of camera in simulation experiments.

f	$x_{p}$	$y_{p}$	**Distortion** **P** **arameters**						
			***K*** **_1_**	***K*** **_2_**	***K*** **_3_**	***P*_1_**	***P*** **_2_**	***A*** **_1_**	***A*** **_2_**
2.9 mm (840.58 pixel)	0.004 mm (1.16 pixel)	0.002 mm (0.58 pixel)	1−5	1−7	3−9	−1−5	−2−7	1−5	2−7
**Pixel Size**	**Image Dimension**		**Image Measurement Random Noise (1** **σ** **)**						
	***x***	***y***							
0.00345 mm	2448 pixel	2048 pixel	0.5 pixel						

**Table 2:** Five different simulation datasets utilized.

Dataset	Location Number (Number of Images)	Usage
a	3, 4 (two images)	Evaluation of the AV-type test-bed
b	1, 2, 3, 4 (four images)	
c	3, 4, 5, 8 (four images)	
d	3, 4, 6, 7 (four images)	
e	All (eight images)	

**Table 3:** Specification of camera in real experiments.

Camera Number	Fish-Eye Lens		Camera Body	
Fish-eye lens Camera 1	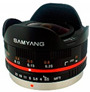 Samyang Fisheye Lens		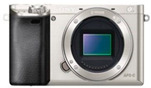 Sony α6000	
	**Projection model**	**Focal length**	**Pixel size**	**Image size (pixel)**
	Equidistant	7.5 mm (1918.16 pixel)	0.00391 mm	6000 × 4000
Fish-eye lens Camera 2	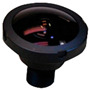 Sunex DSL315		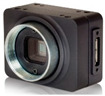 Chameleon3 USB3 5.0 MP	
	**Projection model**	**Focal length**	**Pixel size**	**Image size (pixel)**
	Equisolid-angle	2.67 mm (773.91 pixel)	0.00345 mm	2448 × 2048

**Table 4:** Configuration of image acquisition and dataset for real experiments.

Dataset	Location Number (Number of Images)	Usage	Configuration of Image Acquisition
A	3, 4 (two images)	Evaluation for the AV-type test-bed	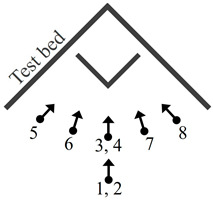
B	1, 2, 3, 4 (four images)		
C	3, 4, 5, 8 (four images)		
D	3, 4, 6, 7 (four images)		
E	All images (eight images)		

**Table 5:** Stability and correlations in simulated datasets.

Projection model	Stability	Dataset	Correlations				
			f−Z0	$x_{p}-X0$	$y_{p}-Y0$	X0−φ	Y0−ω
Equidistant	Stable	a	0.91	0.18	0.21	0.40	0.50
		b	0.83	0.18	0.15	0.45	0.61
		c	0.49	0.09	0.17	0.34	0.49
		d	0.80	0.20	0.28	0.40	0.57
		e	0.53	0.13	0.15	0.44	0.64
		mean	0.71	0.15	0.19	0.41	0.56
Equisolid-angle	Stable	a	0.93	0.19	0.16	0.47	0.52
		b	0.83	0.17	0.12	0.50	0.63
		c	0.49	0.07	0.14	0.40	0.49
		d	0.79	0.19	0.23	0.49	0.60
		e	0.52	0.11	0.14	0.50	0.65
		mean	0.71	0.15	0.16	0.47	0.58
Orthogonal	Stable	a	0.90	0.16	0.10	0.56	0.64
		b	0.81	0.15	0.11	0.60	0.68
		c	0.64	0.08	0.12	0.45	0.54
		d	0.68	0.18	0.14	0.62	0.68
		e	0.58	0.12	0.14	0.59	0.70
		mean	0.72	0.14	0.12	0.57	0.65
Stereographic	Stable	a	0.90	0.14	0.20	0.33	0.47
		b	0.82	0.17	0.16	0.37	0.54
		c	0.53	0.09	0.15	0.27	0.45
		d	0.80	0.20	0.29	0.31	0.50
		e	0.55	0.14	0.16	0.37	0.58
		mean	0.72	0.15	0.19	0.33	0.51

**Table 6:** Absolute error of principal point coordinates (pixel) in simulation experiments.

Projection Model	Dataset					
	a	b	c	d	e	Mean
Equidistant	0.21	0.16	0.14	0.15	0.05	0.14
Equisolid-angle	0.14	0.16	0.11	0.14	0.15	0.14
Orthogonal	0.16	0.12	0.11	0.20	0.12	0.14
Stereographic	0.23	0.34	0.26	0.09	0.23	0.23

**Table 7:** Absolute error of focal length (pixel) in simulation experiments.

Projection Model	Dataset					
	a	b	c	d	e	Mean
Equidistant	0.56	0.10	0.15	0.29	0.13	0.25
Equisolid-angle	0.29	0.03	0.03	0.25	0.09	0.14
Orthogonal	0.32	0.31	0.37	0.24	0.13	0.27
Stereographic	0.09	0.07	0.23	0.14	0.00	0.11

**Table 8:** RMSE value of lens distortion (pixel) in simulation experiments.

Projection Model	Dataset					
	a	b	c	d	e	Mean
Equidistant	0.68	0.29	0.27	0.31	0.20	0.35
Equisolid-angle	0.37	0.12	0.13	0.21	0.07	0.18
Orthogonal	0.18	0.25	0.29	0.26	0.08	0.21
Stereographic	0.41	0.19	0.22	0.26	0.10	0.24

**Table 9:** Root Mean Square residuals (RMS-residuals) of Interior Orientation Parameters (IOPs) (pixel) in simulation experiments.

Projection Model	Dataset					
	a	b	c	d	e	Mean
Equidistant	0.46	0.30	0.25	0.25	0.17	0.29
Equisolid-angle	0.20	0.20	0.19	0.14	0.16	0.18
Orthogonal	0.22	0.10	0.14	0.18	0.14	0.16
Stereographic	0.43	0.48	0.33	0.32	0.33	0.38

**Table 10:** Stability and correlations in real datasets.

Camera	Stability	Dataset	Correlations				
			f−Z0	$x_{p}-X0$	$y_{p}-Y0$	X0−φ	Y0−ω
Fish-eye Lens Camera 1 (Equidistant)	Stable	A	0.95	0.11	0.30	0.31	0.36
		B	0.77	0.19	0.32	0.42	0.22
		C	0.88	0.22	0.40	0.19	0.22
		D	0.93	0.15	0.37	0.29	0.36
		E	0.59	0.58	0.49	0.33	0.38
		mean	0.82	0.25	0.38	0.31	0.31
Fish-eye Lens Camera 2 (Equisolid-angle)	Stable	A	0.93	0.07	0.08	0.45	0.52
		B	0.90	0.05	0.06	0.54	0.67
		C	0.70	0.14	0.22	0.38	0.39
		D	0.80	0.05	0.20	0.49	0.56
		E	0.56	0.50	0.47	0.50	0.55
		mean	0.78	0.16	0.21	0.47	0.54

**Table 11:** Estimated values and standard deviations of principal point coordinates and focal length in real experiments.

Camera	Dataset	Estimated Value (Pixel)			Standard Deviation (Pixel)		
		$x_{p}$	$y_{p}$	f	$x_{p}$	$y_{p}$	f
Fish-eye Lens Camera 1 (equidistant)	A	−4.82	27.39	1910.53	0.32	0.17	0.48
	B	−4.10	27.70	1910.90	0.38	0.23	0.39
	C	−3.91	26.96	1910.79	0.33	0.21	0.35
	D	−4.45	27.33	1910.65	0.28	0.16	0.42
	E	−4.15	27.32	1910.58	0.27	0.20	0.42
Fish-eye Lens Camera 2 (equisolid-angle)	A	−10.06	−32.21	777.56	0.07	0.07	0.22
	B	−10.07	−32.18	777.78	0.06	0.06	0.16
	C	−10.17	−32.24	777.87	0.07	0.07	0.15
	D	−10.04	−31.96	777.66	0.06	0.07	0.13
	E	−10.09	−32.15	777.95	0.09	0.10	0.23

**Table 12:** Estimated values and standard deviations of lens distortion parameters in real experiments (camera 1).

	Dataset	*K*_1_	*K*_2_	*K*_3_	*P*_1_	*P*_2_	*A*_1_	*A*_2_
Estimated Value	A	$-4.28^{-4}$	$9.81^{-7}$	$-2.39^{-8}$	$1.02^{-5}$	$3.49^{-6}$	$3.46^{-4}$	$2.19^{-4}$
	B	$-4.28^{-4}$	$8.18^{-7}$	$-2.20^{-8}$	$1.14^{-5}$	$3.19^{-6}$	$3.46^{-4}$	$2.19^{-4}$
	C	$-4.28^{-4}$	$9.52^{-7}$	$-2.36^{-8}$	$1.02^{-5}$	$3.49^{-6}$	$3.46^{-4}$	$2.19^{-4}$
	D	$-4.28^{-4}$	$9.10^{-7}$	$-2.31^{-8}$	$1.02^{-5}$	$3.49^{-6}$	$3.46^{-4}$	$2.19^{-4}$
	E	$-4.28^{-4}$	$1.01^{-6}$	$-2.40^{-8}$	$1.02^{-5}$	$3.49^{-6}$	$3.46^{-4}$	$2.19^{-4}$
Standard deviation	A	$6.27^{-5}$	$4.22^{-7}$	$1.10^{-8}$	$5.70^{-6}$	$1.92^{-6}$	$8.74^{-5}$	$7.22^{-5}$
	B	$3.42^{-5}$	$2.62^{-7}$	$5.51^{-9}$	$3.07^{-6}$	$1.05^{-6}$	$7.26^{-5}$	$5.69^{-5}$
	C	$1.31^{-5}$	$1.81^{-7}$	$3.07^{-9}$	$2.23^{-6}$	$1.08^{-6}$	$3.80^{-5}$	$4.16^{-5}$
	D	$5.13^{-5}$	$2.09^{-7}$	$4.15^{-9}$	$3.57^{-6}$	$1.29^{-6}$	$8.64^{-5}$	$5.91^{-5}$
	E	$1.15^{-5}$	$1.82^{-7}$	$1.92^{-9}$	$1.93^{-6}$	$8.39^{-7}$	$5.53^{-5}$	$3.72^{-5}$

**Table 13:** Estimated values and standard deviations of lens distortion parameters in real experiments (camera 2).

	Dataset	*K*_1_	*K*_2_	*K*_3_	*P*_1_	*P*_2_	*A*_1_	*A*_2_
Estimated Value	A	$-4.17^{-3}$	$3.36^{-4}$	$-3.97^{-5}$	−$9.22^{-5}$	$6.83^{-5}$	$1.86^{-4}$	−$8.84^{-5}$
	B	$-4.17^{-3}$	$3.36^{-4}$	−$3.99^{-5}$	$-9.22^{-5}$	$6.83^{-5}$	$1.86^{-4}$	−$8.83^{-5}$
	C	$-4.17^{-3}$	$3.36^{-4}$	−$4.03^{-5}$	$-9.23^{-5}$	$6.83^{-5}$	$1.86^{-4}$	−$8.83^{-5}$
	D	$-4.17^{-3}$	$3.37^{-4}$	−$3.99^{-5}$	$-9.22^{-5}$	$6.83^{-5}$	$1.86^{-4}$	−$8.83^{-5}$
	E	$-4.17^{-3}$	$3.36^{-4}$	−$4.02^{-5}$	$-9.22^{-5}$	$6.83^{-5}$	$1.86^{-4}$	$-8.83^{-5}$
Standard deviation	A	$6.25^{-5}$	$8.41^{-5}$	$7.41^{-6}$	$2.49^{-5}$	$2.66^{-5}$	$7.99^{-5}$	$3.44^{-5}$
	B	$2.92^{-4}$	$5.72^{-5}$	$3.59^{-6}$	$1.38^{-5}$	$1.71^{-5}$	$6.69^{-5}$	$1.50^{-5}$
	C	$2.08^{-4}$	$3.36^{-5}$	$2.01^{-6}$	$1.29^{-5}$	$1.37^{-5}$	$5.20^{-5}$	$1.06^{-5}$
	D	$4.17^{-4}$	$8.08^{-5}$	$4.79^{-6}$	$1.84^{-5}$	$1.98^{-5}$	$4.83^{-5}$	$1.68^{-5}$
	E	$1.67^{-4}$	$2.69^{-5}$	$1.61^{-6}$	$9.22^{-6}$	$6.83^{-6}$	$3.53^{-5}$	$9.71^{-6}$

**Table 14:** RMS-residuals of IOPs in real experiments.

Camera	Dataset					
	A	B	C	D	E	Mean
Fish-eye Lens Camera 1 (Equisolid-angle)	0.27	0.20	0.23	0.28	0.37	0.27
Fish-eye Lens Camera 2 (Equidistant)	0.34	0.31	0.39	0.36	0.35	0.35

**Table 15:** Comparison of absolute error of principal point coordinates (pixel).

Projection Model	Dataset									
	AV-Type Test-Bed						V-Type Test-Bed (Choi et al. [50])			
	a (2)	b (4)	c (4)	d (4)	e (8)	Mean	1 (10)	2 (10)	3 (14)	Mean
Equidistant	0.21	0.16	0.14	0.15	0.05	0.14	0.40	0.78	0.71	0.63
Equisolid-angle	0.14	0.16	0.11	0.14	0.15	0.14	0.56	0.32	0.46	0.45
Orthogonal	0.16	0.12	0.11	0.20	0.12	0.14	0.26	0.41	0.11	0.26
Stereographic	0.23	0.34	0.26	0.09	0.23	0.23	0.41	0.37	0.18	0.32

**Table 16:** Comparison of absolute error of focal length (pixel).

Projection Model	Dataset									
	AV-Type Test-Bed						V-Type Test-Bed (Choi et al. [50])			
	a (2)	b (4)	c (4)	d (4)	e (8)	Mean	1 (10)	2 (10)	3 (14)	Mean
Equidistant	0.56	0.10	0.15	0.29	0.13	0.25	1.97	0.04	0.04	0.68
Equisolid-angle	0.29	0.03	0.03	0.25	0.09	0.14	1.25	0.22	0.46	0.64
Orthogonal	0.32	0.31	0.37	0.24	0.13	0.27	1.91	0.58	0.16	0.88
Stereographic	0.09	0.07	0.23	0.14	0.00	0.11	0.61	0.96	0.70	0.76

**Table 17:** Comparison of RMSE values of lens distortion (pixel).

Projection Model	Dataset									
	AV-Type Test-Bed						V-Type Test-Bed (Choi et al. [50])			
	a (2)	b (4)	c (4)	d (4)	e (8)	Mean	1 (10)	2 (10)	3 (14)	Mean
Equidistant	0.68	0.29	0.27	0.31	0.20	0.35	7.48	0.18	0.15	2.60
Equisolid-angle	0.37	0.12	0.13	0.21	0.07	0.18	2.47	0.34	0.39	1.07
Orthogonal	0.18	0.25	0.29	0.26	0.08	0.21	1.21	0.32	0.08	0.54
Stereographic	0.41	0.19	0.22	0.26	0.10	0.24	3.12	1.50	0.75	1.79

**Table 18:** Comparison of RMS-residuals of IOPs (pixel).

Projection Model	Dataset									
	AV-Type Test-Bed						V-Type Test-Bed (Choi et al. [50])			
	a (2)	b (4)	c (4)	d (4)	e (8)	Mean	1 (10)	2 (10)	3 (14)	Mean
Equidistant	0.46	0.30	0.25	0.25	0.17	0.29	6.12	0.85	0.70	2.56
Equisolid-angle	0.20	0.20	0.19	0.14	0.16	0.18	1.63	0.28	0.39	0.77
Orthogonal	0.22	0.10	0.14	0.18	0.14	0.16	0.38	0.35	0.11	0.28
Stereographic	0.43	0.48	0.33	0.32	0.33	0.38	2.45	0.78	0.61	1.28

**Table 19:** Comparison with other self-calibration results using real datasets.

Approaches	Projection Model	Number of Used Images	Standard Deviation (Pixel)			RMS-Residuals of IOPs (Pixel)
			$x_{p}$	$y_{p}$	f	
Proposed	Equidistant	2–8	0.27–0.38	0.16–0.23	0.35–0.48	0.20–0.37
	Equisolid-angle	2–8	0.06–0.09	0.06–0.10	0.13–0.23	0.31–0.39
Marcato et al. [12]	Stereographic	43	0.20	0.20	0.19	0.51
Sahin [36] (used 2 cameras)	Equidistant	13	0.96/2.84	0.89/2.85	0.75/1.15	0.60/0.71
Schneider et al. [38]	Equisolid-angle	9	0.78	3.14	0.95	0.30

## Data Availability

Data sharing not applicable.
